# Supplementary material for: Genetic control of thermomorphogenesis in tomato inflorescences
Source: Nat Commun. 2024 Feb 17;15:1472. doi: 10.1038/s41467-024-45722-0 (PMC10874430; doi:10.1038/s41467-024-45722-0)
Supplement: Supplementary file 1 — Supplementary Information [file 41467_2024_45722_MOESM1_ESM.pdf]

Genetic control of thermomorphogenesis in tomato inflorescences

Sun *et al.*

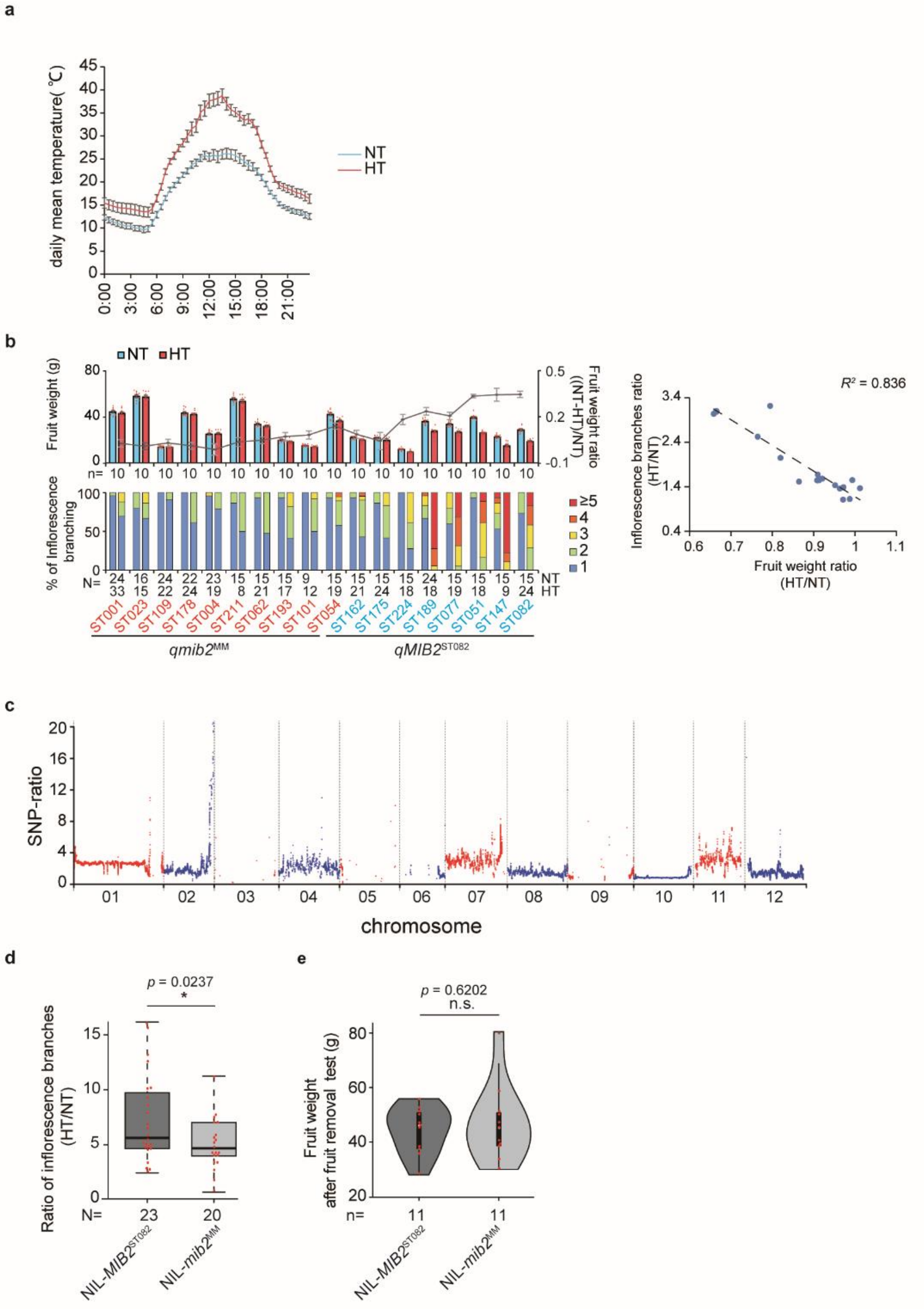

Supplementary Fig. 1 Analysis of the effect of *qMIB2* locus on reproductive development.
a, Mean temperature at different time points of the day under normal temperature (NT) and high temperature (HT) conditions in the field. **b,** Fruit weight and inflorescence branch number of a subset of the RIL population derived from a cross between CC and MM under NT and HT conditions (left), and correlation between the ratio of fruit weight and inflorescence branch number between NT and HT conditions (right). Red words represent lines carrying the $qmib2^{MM}$ allele, blue words represent lines carrying the $qMIB2^{ST082}$ allele. **c,** SNP-ratios between different pools of segregating phenotypic classes. **d,** Inflorescence branches ratios of NILs between HT and NT conditions. **e,** Fruit weight of NILs after the fruit removal test (only 5 fruits were kept for each inflorescence). Data in (**d, e**) were compared by two-tailed Student's t-test, *$p < 0.05$, n.s. no significance difference ($p > 0.05$). Box edges in (**d, e**) represent the 0.25 and 0.75 quantiles, and the bold lines indicate median values. Whiskers indicate 1.5 times the interquartile range, the bar ranges the minimum to maximum observations. N in (**b, d**) = number of inflorescences. n in (**b, e**) = number of plants. Source data are provided as a Source Data file.

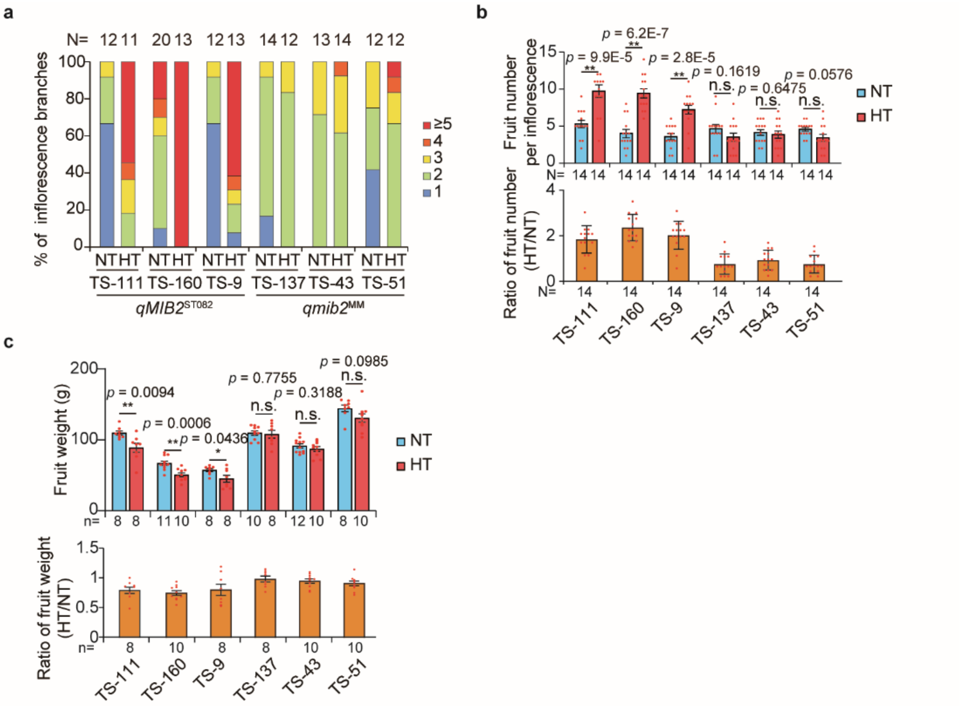

Supplementary Fig. 2 Analysis of the effect of *qMIB2* in natural population.
a, Inflorescence branch number of three tomato accessions with *qMIB2*^{ST082} or *qmib2*^{MM} under NT and HT conditions. N = number of inflorescences. **b,** Fruit number of three tomato accessions with *qMIB2*^{ST082} or *qmib2*^{MM} under NT and HT conditions. **c,** Fruit weight of three tomato accessions with *qMIB2*^{ST082} or *qmib2*^{MM} under NT and HT conditions. Data in (**b, c**) were compared by two-tailed Student's *t*-test, $^{**}p < 0.01$, $^{*}p < 0.05$, n.s. no significance difference ($p > 0.05$). Values are means ± SEM. N in (**b**) = number of inflorescences. n in (**c**) = number of plants. Source data are provided as a Source Data file.

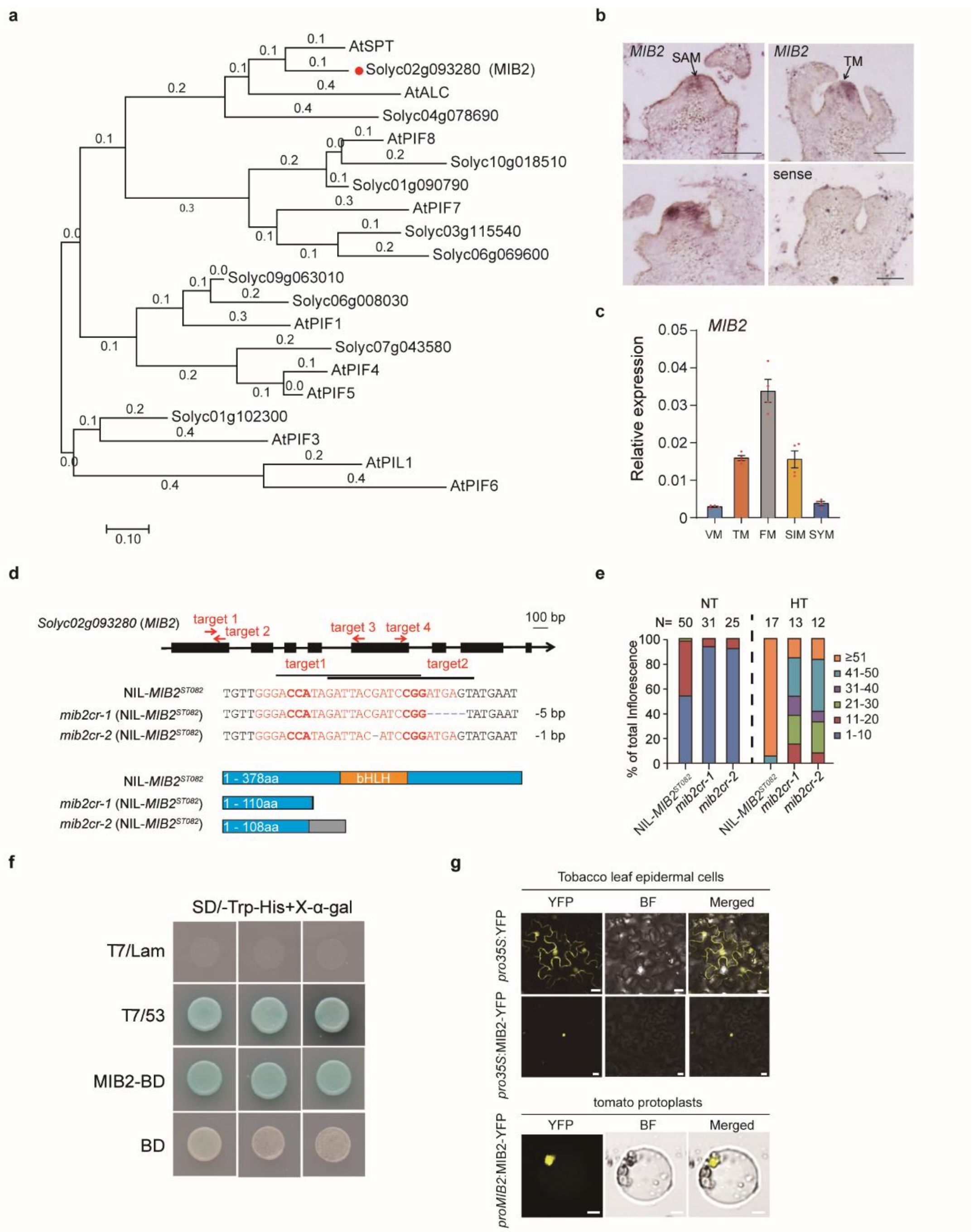

Supplementary Fig. 3 *MIB2* encodes a bHLH transcription activator.
a, Phylogenetic tree of the of the PIFs subfamily members belonging to the bHLH transcription factors in Arabidopsis (*At*) and tomato by maximum likelihood method based on the JTT matrix-based model. The scale bar represents 10 amino acid replacements per 100 positions. **b,** *In situ* hybridization of *MIB2* in different meristematic tissues of ST082. SAM, shoot apical meristem; TM, transition meristem; SIM, sympodial inflorescence meristem; FM, floral meristem. Scale bars=100 μm. 4 independent experiments were performed. **c,** Relative *MIB2* expression in different meristematic tissues of ST082. VM, vegetable meristem. n = 4 biologically independent replicates. **d,** Genotype summary of *mib2cr* mutants. The red font indicates the target sequences. The blue dashed lines indicate the deleted sequences in the mutants. The bold font indicates the protospacer-adjacent motif (PAM). Premature stop codon in these mib2cr mutants resulted in a truncated protein of 110 amino acids and 108 amino acids in length, respectively. The orange box represents the bHLH domain. **e,** The quantification data of inflorescence branch number of the NIL-*MIB2*^{ST082} plants and the *mib2cr* mutants. The % of total inflorescences represents the proportion of the number of inflorescences within a certain range of inflorescence branches to a total number of inflorescences. N = number of inflorescences.**f,** The transcriptional activation activity of MIB2 in yeast cells. T7/Lam is negative control, T7/53 is positive control. 3 independent experiments were performed.**g,** Subcellular localization of MIB2-YFP in tobacco leaf epidermal cells and tomato protoplasts. Six independent experiments were performed. Source data are provided as a Source Data file.

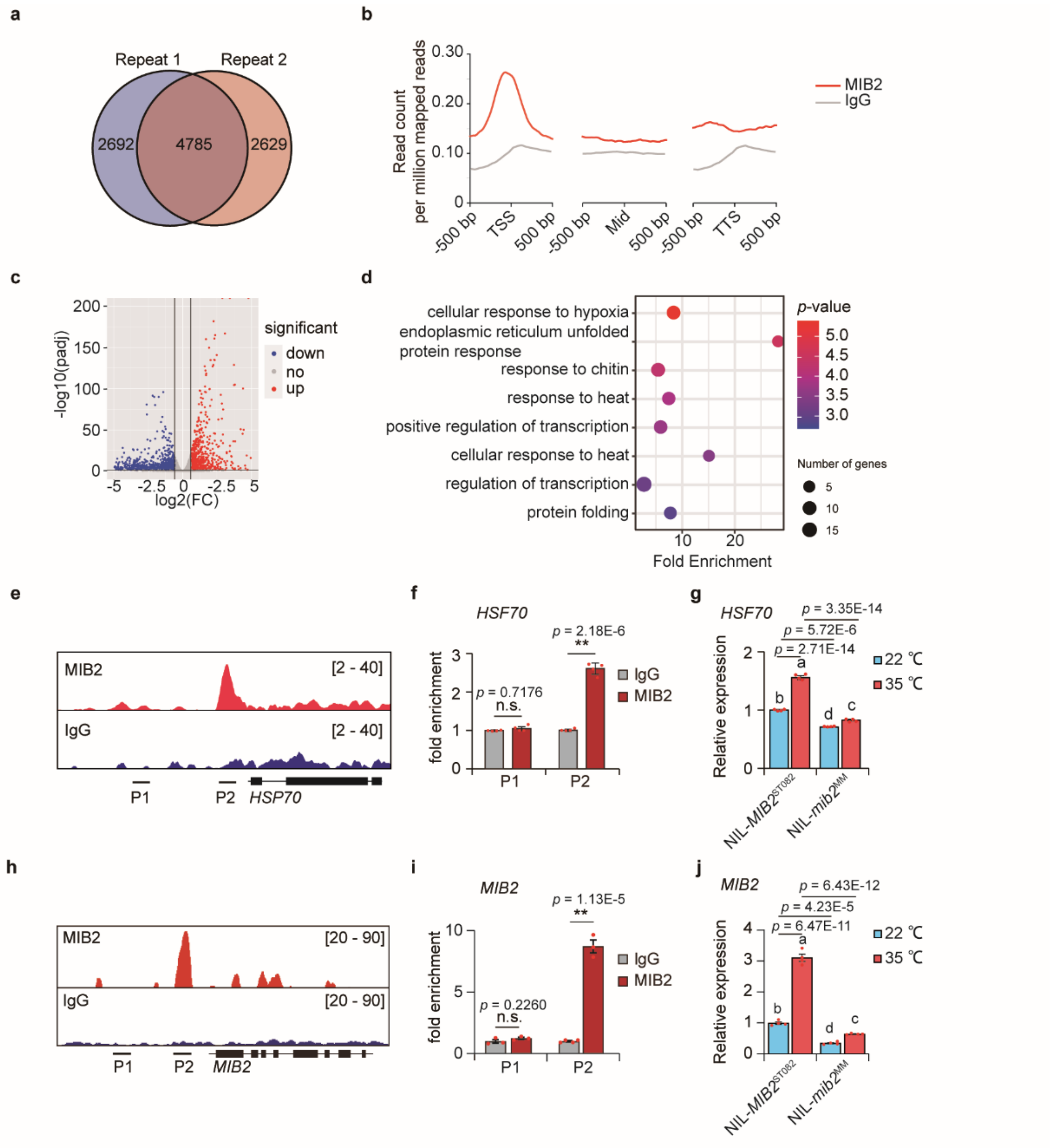

Supplementary Fig. 4 ChIP-seq and RNA-seq analysis of the binding sites of MIB2.
a, Venn diagram showing the number of binding sites by MIB2 in two biological replicates. **b,** Metaplot profiles of MIB2 signals on target genes based on ChIP-seq data. The distribution of the MIB2 signal for each gene in fixed 3-kb regions around the transcription start site (TSS), transcription termination site (TTS), and the center of the gene was averaged and plotted. IgG was used as the control. **c,** Volcano plot showing differential gene expression between NIL-*MIB2*^{ST082} and NIL-*mib2*^{MM} as determined by RNA-seq. Blue dots indicate downregulated genes in NIL-*mib2*^{MM}, and red dots indicate upregulated genes in NIL-*mib2*^{MM} compared with NIL-*MIB2*^{ST082}. **d,** Gene ontology (GO) term enrichment analysis of the overlapping gene between the putative MIB2-bound genes identified by ChIP-seq and differentially regulated genes in NIL-*mib2*^{MM} relative to NIL-*MIB2*^{ST082} as identified by RNA-seq. The top 8 most highly enriched GO terms in the biological processes category with *p*-values from the statistical over-representation test are shown. **e,** Chromatin binding profiles of MIB2 at the *HSP70* promoters. The short black lines labeled P1 and P2 represent the regions used for ChIP-qPCR. **f,** ChIP-qPCR analysis of the bound regions in the *MIB2* promoters. P1 and P2 represent the regions used for ChIP-qPCR. Input was used as a negative control. The DNA fragment of the *ACTIN* (*Solyc03g078400*) 3' intergenic region was used as an internal control. **g,** Expression levels of *HSP70* in meristematic tissues of NIL-*MIB2*^{ST082} and NIL-*mib2*^{MM} plants under NT and HT conditions. *UBIQUITIN3* (*Solyc01g056940*) was used as the reference transcript. **h,** Chromatin binding profiles of MIB2 at the *MIB2* promoters. The short black lines labeled P1 and P2 represent the regions used for ChIP-qPCR. **i,** ChIP-qPCR analysis of the bound regions in the *MIB2* promoters. P1 and P2 represent the regions used for ChIP-qPCR. Input was used as a negative control. The DNA fragment of the *ACTIN* (*Solyc03g078400*) 3' intergenic region was used as an internal control. **j,** Expression levels of *MIB2* in meristematic tissues of NIL-*MIB2*^{ST082} and NIL-*mib2*^{MM} plants under NT and HT conditions. *UBIQUITIN3* (*Solyc01g056940*) was used as the reference transcript. Data in (**f, i**) were compared by two-tailed Student's t-test, **, $p < 0.01$; n.s. no significant difference ($p > 0.05$). Different letters in (**g, j**) indicate a significant difference ($p < 0.05$) based on the one-way ANOVA followed by Tukey's multiple comparisons test. Values in (**f, g, i, j**) are means ± SEM (n ≥ 3 biologically independent replicates). Source data are provided as a Source Data file.

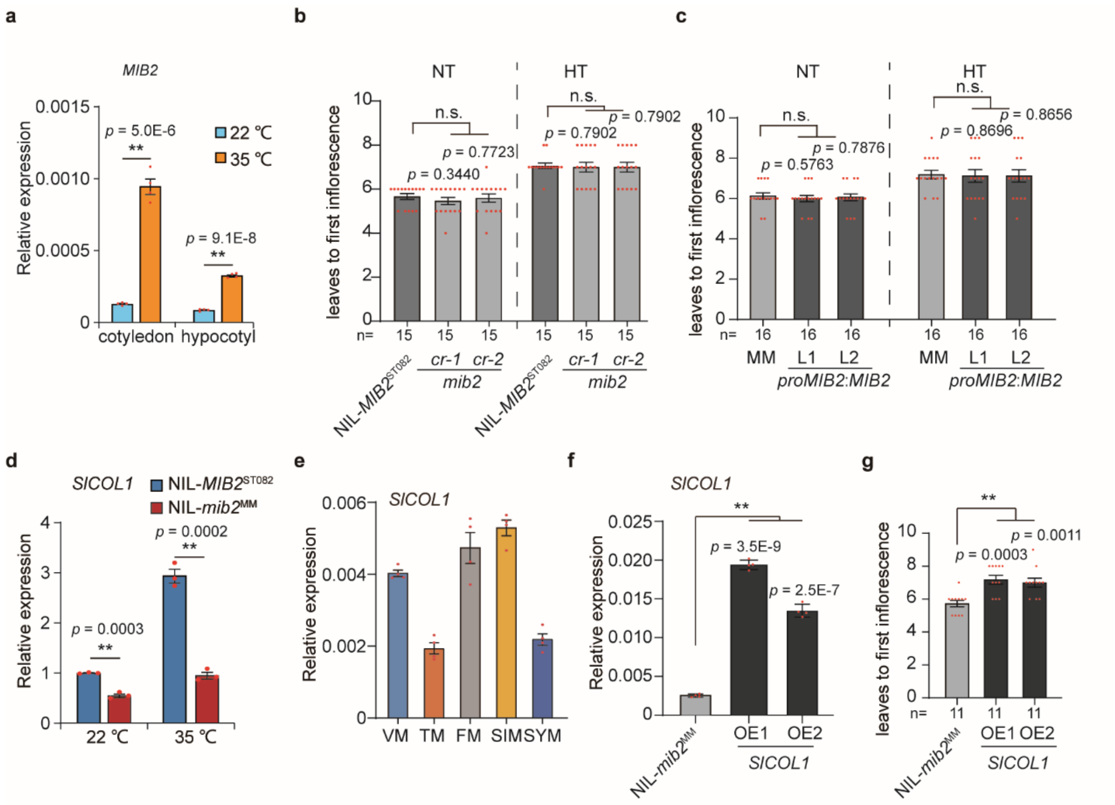

Supplementary Fig. 5 Flowering time in *MIB2* or *SlCOL1* transgenes lines.
a, Relative transcription levels of *MIB2* in cotyledon and hypocotyl of NIL-*MIB2*^{ST082} plants at different temperature. **b, c,** Number of leaves before the first inflorescence of the NIL-*MIB2*^{ST082} and *mib2cr* mutants (**b**) or the MM and *proMIB2*:*MIB2-YFP-HA* plants (**c**) under NT and HT conditions. **d,** Relative transcription levels of *SlCOL1* in NIL-*MIB2*^{ST082} and NIL-*mib2*^{MM} meristematic tissues at NT and HT. **e,** Relative transcription levels of *SlCOL1* in different meristematic tissues of ST082. VM, vegetable meristem; TM, transition meristem; SIM, sympodial inflorescence meristem; FM, floral meristem. **f,** Relative transcription levels of *SlCOL1* in meristematic tissues of *SlCOL1* overexpression lines. **g,** Number of leaves before first inflorescence of the NIL-*mib2*^{MM} and *SlCOL1*-OE plants. Data in (**a - d, f,g**) were compared by two-tailed Student's *t*-test, $^{**}p < 0.01$, n.s. no significance difference ($p > 0.05$). Values are means ± SEM. n in (**a, e, f**) = 4 biologically independent replicates. n in (**d**) = 3 biologically independent replicates. n in (**b, c, g**) = number of plants. Source data are provided as a Source Data file.

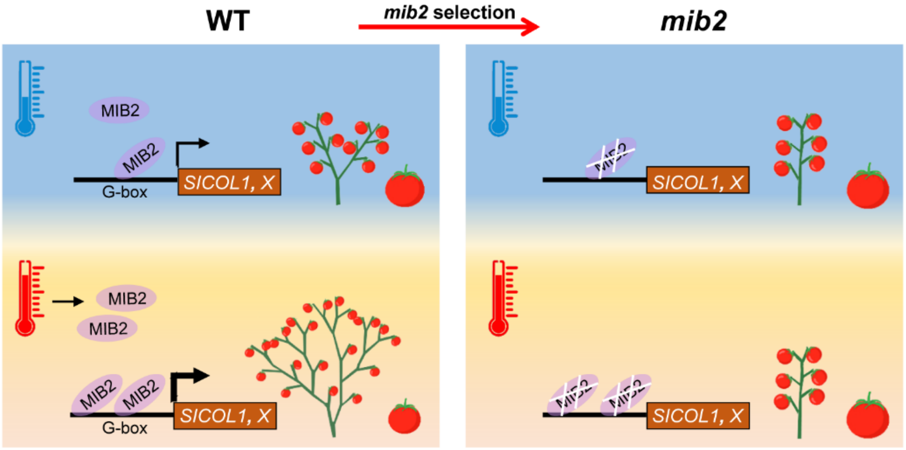

Supplementary Fig. 6 Working model of the role of the human selection target *MIB2* in regulating inflorescence branching under NT and HT conditions.

When conditions are NT (about 25 °C), *MIB2* expression level is normal, which *SlCOL1* expression level is slightly induced, which inflorescence branching is complex and the fruit weight is low. When tomato meristemic tissues are under HT conditions (more than 35 °C), *MIB2* expression level are significantly elevated, while MIB2 protein accumulates at the promoter of *SlCOL1*. As a result, *SlCOL1* expression is sharply increased, leading to the markedly increased of inflorescence branches, and the dramatically decreased of fruit weight. In some big-fruited *S. lycopersicum* accessions, the *mib2* allele may be human selected, which *SlCOL1* expression level is reduced without activator MIB2. So that whether under NT or HT conditions, the inflorescence branching can be stable and simple, and the fruit weight is more stable and larger.
