# Supplementary material for: Genetic control of thermomorphogenesis in tomato inflorescences
Source: Nat Commun. 2024 Feb 17;15:1472. doi: 10.1038/s41467-024-45722-0 (PMC10874430; doi:10.1038/s41467-024-45722-0)
Supplement: Supplementary file 3 — Description of Additional Supplementary Files [file 41467_2024_45722_MOESM3_ESM.pdf]

Description of Additional Supplementary Files

Supplementary Data 1. List of ChIP-Seq peaks bound by MIB2.

Supplementary Data 2. The genes expression in each sample by RNA-seq.

Supplementary Data 3. List of primers used in this work.
