# Supplementary material for: Genetic control of thermomorphogenesis in tomato inflorescences
Source: Nat Commun. 2024 Feb 17;15:1472. doi: 10.1038/s41467-024-45722-0 (PMC10874430; doi:10.1038/s41467-024-45722-0)
Supplement: Supplementary file 7 — Reporting Summary [file 41467_2024_45722_MOESM7_ESM.pdf]

nature portfolio

Corresponding author(s): Xia Cui

Last updated by author(s): Jan 29, 2024

Reporting Summary

Nature Portfolio wishes to improve the reproducibility of the work that we publish. This form provides structure for consistency and transparency in reporting. For further information on Nature Portfolio policies, see our Editorial Policies and the Editorial Policy Checklist.

Statistics

For all statistical analyses, confirm that the following items are present in the figure legend, table legend, main text, or Methods section.

n/a	Confirmed	
☐	☒	The exact sample size (*n*) for each experimental group/condition, given as a discrete number and unit of measurement
☐	☒	A statement on whether measurements were taken from distinct samples or whether the same sample was measured repeatedly
☐	☒	The statistical test(s) used AND whether they are one- or two-sided<br>*Only common tests should be described solely by name; describe more complex techniques in the Methods section.*
☒	☐	A description of all covariates tested
☐	☒	A description of any assumptions or corrections, such as tests of normality and adjustment for multiple comparisons
☐	☒	A full description of the statistical parameters including central tendency (e.g. means) or other basic estimates (e.g. regression coefficient) AND variation (e.g. standard deviation) or associated estimates of uncertainty (e.g. confidence intervals)
☐	☒	For null hypothesis testing, the test statistic (e.g. *F*, *t*, *r*) with confidence intervals, effect sizes, degrees of freedom and *P* value noted<br>*Give P values as exact values whenever suitable.*
☒	☐	For Bayesian analysis, information on the choice of priors and Markov chain Monte Carlo settings
☒	☐	For hierarchical and complex designs, identification of the appropriate level for tests and full reporting of outcomes
☒	☐	Estimates of effect sizes (e.g. Cohen's *d*, Pearson's *r*), indicating how they were calculated

Our web collection on statistics for biologists contains articles on many of the points above.

Software and code

Policy information about availability of computer code

Data collection	No software was used in data collection
Data analysis	STAR version 2.5.3, DEGseq and FeatureCounts version 1.5.3 for RNA-Seq anaysis. Bowtie2 version 2.3.5, SAMtools version 1.9, MACS2 version 2.1, MEME suite version 5.5.4 and DeepTools version 3.3.0 for ChIP-Seq analysis.

For manuscripts utilizing custom algorithms or software that are central to the research but not yet described in published literature, software must be made available to editors and reviewers. We strongly encourage code deposition in a community repository (e.g. GitHub). See the Nature Portfolio guidelines for submitting code & software for further information.

Data

Policy information about availability of data

All manuscripts must include a data availability statement. This statement should provide the following information, where applicable:

- Accession codes, unique identifiers, or web links for publicly available datasets
- A description of any restrictions on data availability
- For clinical datasets or third party data, please ensure that the statement adheres to our policy

The reference genome of Heinz 1706 (build SL4.0 and genome annotation ITAG4.0) was download from Sol Genomics Network (SGN, https://solgenomics.net/). The RNA - seq and ChIP - seq datasets in this study have been deposited in the NCBI Sequence Read Archive (SRA) under BioProject accession PRJNA943168 and SRR12756256. Reviewer link: https://www.ncbi.nlm.nih.gov/sra/PRJNA943168; https://www.ncbi.nlm.nih.gov/sra/SRR12756256.

Research involving human participants, their data, or biological material

Policy information about studies with human participants or human data. See also policy information about sex, gender (identity/presentation), and sexual orientation and race, ethnicity and racism.

Reporting on sex and gender	Not applicable
Reporting on race, ethnicity, or other socially relevant groupings	Not applicable
Population characteristics	Not applicable
Recruitment	Not applicable
Ethics oversight	Not applicable

Note that full information on the approval of the study protocol must also be provided in the manuscript.

Field-specific reporting

Please select the one below that is the best fit for your research. If you are not sure, read the appropriate sections before making your selection.

☒ Life sciences ☐ Behavioural & social sciences ☐ Ecological, evolutionary & environmental sciences

For a reference copy of the document with all sections, see nature.com/documents/nr-reporting-summary-flat.pdf

Life sciences study design

All studies must disclose on these points even when the disclosure is negative.

Sample size	Details of biological/technical replicates are provided in the manuscript. The sample size was as big as feasible for our experimental approaches. More than 5 plants were used for phenotyping analysis. 5–8 inflorescences or fruits were evaluated per plant.
Data exclusions	No data was excluded.
Replication	All growth measurements were highly reproducible. All the experiments were conducted at least three times to verify the reproducibility. The replication of experiments in this study were described in the legend. All attempts at replication were successful.
Randomization	All plants were randomized in the growth chambers to avoid edge effects. Inflorescence branching used for detection. Fruit weight used for measurements were collected in random at same ripening stage.
Blinding	All plants were randomized and simple size was reasonable. Thus, blinding is not possible.

Reporting for specific materials, systems and methods

We require information from authors about some types of materials, experimental systems and methods used in many studies. Here, indicate whether each material, system or method listed is relevant to your study. If you are not sure if a list item applies to your research, read the appropriate section before selecting a response.

Materials & experimental systems

n/a	Involved in the study	
☐	☒	Antibodies
☒	☐	Eukaryotic cell lines
☒	☐	Palaeontology and archaeology
☒	☐	Animals and other organisms
☒	☐	Clinical data
☒	☐	Dual use research of concern
☐	☒	Plants

Methods

n/a	Involved in the study	
☐	☒	ChIP-seq
☒	☐	Flow cytometry
☒	☐	MRI-based neuroimaging

Antibodies

Antibodies used	anti-HA (Sigma-Aldrich, H6908, 1:2000); anti-Actin (Sigma-Aldrich, A0480, 1:2000); Goat Anti-Rabbit IgG Antibody, HRP-conjugated (Sigma-Aldrich, 12-348, 1:30000); Goat Anti-Mouse IgG Antibody, HRP conjugate (Sigma-Aldrich, 12-349, 1:30000) were used for WB

	experiment; anti-HA (Sigma-Aldrich, H6908, 5 ug/sample) and anti-IgG (Sigma-Aldrich, R2655, 5 ug/sample) were used for ChIP experiment.
Validation	anti-HA (https://www.sigmaaldrich.cn/CN/zh/product/sigma/h6908); anti-Actin (https://www.sigmaaldrich.cn/CN/zh/product/sigma/a0480); Goat Anti-Rabbit IgG Antibody, HRP-conjugated (https://www.sigmaaldrich.cn/CN/zh/product/mm/12348); Goat Anti-Mouse IgG Antibody, HRP conjugate (Shttps://www.sigmaaldrich.cn/CN/zh/product/mm/12349) ;anti-IgG (https://www.sigmaaldrich.cn/CN/zh/product/sigma/r2655) .

Dual use research of concern

Policy information about dual use research of concern

Hazards

Could the accidental, deliberate or reckless misuse of agents or technologies generated in the work, or the application of information presented in the manuscript, pose a threat to:

No	Yes	
☒	☐	Public health
☒	☐	National security
☒	☐	Crops and/or livestock
☒	☐	Ecosystems
☒	☐	Any other significant area

Experiments of concern

Does the work involve any of these experiments of concern:

No	Yes	
☒	☐	Demonstrate how to render a vaccine ineffective
☒	☐	Confer resistance to therapeutically useful antibiotics or antiviral agents
☒	☐	Enhance the virulence of a pathogen or render a nonpathogen virulent
☒	☐	Increase transmissibility of a pathogen
☒	☐	Alter the host range of a pathogen
☒	☐	Enable evasion of diagnostic/detection modalities
☒	☐	Enable the weaponization of a biological agent or toxin
☒	☐	Any other potentially harmful combination of experiments and agents

Plants

Seed stocks	The source of all seed stocks can be found in Zhang, S.B. et al.,2018, Lin, T. et al., 2016. The RILs were derived by single seed descent from a cross between the cultivated tomato S. lycopersicum cv. Moneymaker and the cherry tomato (LA1310).
Novel plant genotypes	All vectors for transgenic were validated by Sanger sequencing (BGI company) and transformed into Agrobacterium strain AGL1 and then were transformed by the leaf disc transformation method. T0 plants' genome DNA was used as template for PCR. For CRISPR lines, the fragment containing the target site was sequenced to identify homozygous genome-edited plants. For complementation and OE lines, detection of T-DNA insertion by electrophoresis.
Authentication	Genomic DNA was extracted from transgenic T0 plants and used as template for PCR. For CRISPR lines, the fragment containing the target site was subjected to Sanger sequencing (BGI company) to identify homozygous genome-edited plants. For complementation and overexpression lines, detection of T-DNA insertion by electrophoresis.

ChIP-seq

Data deposition

☒ Confirm that both raw and final processed data have been deposited in a public database such as GEO.

☐ Confirm that you have deposited or provided access to graph files (e.g. BED files) for the called peaks.

Data access links<br>*May remain private before publication.*	https://dataview.ncbi.nlm.nih.gov/object/PRJNA943168?reviewer=ego6n7bgto2que4mmrr0iidtvk<br>https://www.ncbi.nlm.nih.gov/sra/SRR12756256
Files in database submission	MIB2_rep1.fastq.gz

Files in database submission	MIB2_rep2.fastq.gz<br>IgG.fastq.gz
Genome browser session (e.g. UCSC)	https://solgenomics.net/jbrowse_solgenomics/?data=data%2Fjson%2FSL4.0&loc=SL4.0ch02%3A52245195..52246112&tracks=DNA&highlight=

Methodology

Replicates	Two replicates used for ChIP-seq
Sequencing depth	For ChIP-seq, more than 30M reads were sequencing depth for each experiment:<br>Repeat1, total reads number: 77393863, uniquely mapped reads number: 25540129, length 150 bp ,Single-end<br>Repeat2, total reads number: 94135458, uniquely mapped reads number: 26539869, length 150 bp, Single-end<br>IgG, total reads number: 68048694, uniquely mapped reads number: 27652642, length 150 bp, Single-end
Antibodies	anti-HA (Sigma-Aldrich, H6908, 5 ug/sample) and anti-IgG (Sigma-Aldrich, R2655, 5 ug/sample) were used for ChIP experiment
Peak calling parameters	Default parameters.
Data quality	4,785 overlapping peaks of two replicates at fold enrichment > 4, q-value < 0.00001.
Software	Bowtie2 version 2.3.5, SAMtools version 1.9, MACS2 version 2.1, MEME suite version 5.5.4 and DeepTools version 3.3.0 for ChIP-Seq analysis.
